# Supplementary material for: Learning structured population models from data with WSINDy
Source: PLoS Comput Biol. 2025 Dec 8;21(12):e1013742. doi: 10.1371/journal.pcbi.1013742 (PMC12685223; doi:10.1371/journal.pcbi.1013742)
Supplement: S1 Appendix — (PDF) [file pcbi.1013742.s001.pdf]

Supporting information: Learning structured population models from data with WSINDy

Simulation details

Due to the histogram-style interpretation of the data, it is natural to use a finite-volume method to construct artificial data for the example problems. To this end, the artificial examples presented in Table 2 are simulated using a minmod flux-limiter discretization which has been previously used and analyzed for a variety of size-structured models [32, 36, 47, 48] and we refer the reader to these works for more details and convergence properties of the scheme. For simplicity, we present the method here for the size-structured population model (2) and remark that the age-structured case follows with $g \equiv \alpha$. Let $\Delta x > 0$ be a given mesh size and let $\Lambda_i := (x_i - \frac{1}{2}\Delta x, x_i + \frac{1}{2}\Delta x]$ represent uniformly placed cells with midpoints $x_i = (i - \frac{1}{2})\Delta x$ with $i = 1, 2, \ldots, I$. We then discretize the initial condition as $n_i^0 := \frac{1}{|\Lambda_i|}\int_{\Lambda_i} n(0, x)\,\mathrm{d}x$ and, through integrating (2) over the domains Λ_i, arrive at the following system of differential equations which describe the evolution of the volumes $n_i(t) = \frac{1}{|\Lambda_i|}\int_{\Lambda_i} n(t, x)\,\mathrm{d}x$,

$$\begin{cases} \dfrac{\mathrm{d}}{\mathrm{d}t} n_i = -\dfrac{1}{\Delta x}[\mathcal{F}_{i+1/2} - \mathcal{F}_{i-1/2}] - d[\vec{n}]_i n_i, & \text{(1a)} \\ g[\vec{n}]_0 n_0 = \Delta x \left(\dfrac{3}{2}\beta[\vec{n}]_1 n_1 + \displaystyle\sum_{i=2}^{I-1} \beta[\vec{n}]_i n_i + \dfrac{1}{2}\beta[\vec{n}]_I n_I \right), & \text{(1b)} \end{cases}$$

where the numerical fluxes $\mathcal{F}_{i+1/2} \approx g[\vec{n}](x_{i+1/2}) n_{i+1/2}$ are given by

$$\mathcal{F}_{i+1/2} := \begin{cases} g[\vec{n}]_i n_i + \frac{1}{2}(g[\vec{n}]_{i+1} - g[\vec{n}]_i) n_i + \frac{1}{2} g[\vec{n}]_i \mathrm{mm}(n_{i+1} - n_i, n_i - n_{i-1}), & i = 2, \ldots, I-2 \\ g[\vec{n}]_i n_i, & i = 0, 1, I-1, I. \end{cases}$$

Here, we denote the minmod function by $\mathrm{mm}(a, b)$ which is given by

$$\mathrm{mm}(a, b) := \frac{\mathrm{sgn}(a) + \mathrm{sgn}(b)}{2} \min(|a|, |b|).$$

In the case of size-structured models, when it is assumed that $g[n](s_{\max}) n(t, s_{\max}) = 0$, then we set $\mathcal{F}_{I+1/2} = 0$. System (1) is then solved using a high-order explicit method; in this work, we used MATLAB's ode45 with default tolerances. All examples were simulated with $I = 30,000$.
