# Supplementary material for: Learning structured population models from data with WSINDy
Source: PLoS Comput Biol. 2025 Dec 8;21(12):e1013742. doi: 10.1371/journal.pcbi.1013742 (PMC12685223; doi:10.1371/journal.pcbi.1013742)
Supplement: S2 Appendix — (PDF) [file pcbi.1013742.s002.pdf]

Supporting information: Learning structured population models from data with WSINDy

Approximation of the Total Population

Suppose we are given noisy time series data of the population number density in the form $n_{k,j} := \varepsilon_{k,j} n^\star_{k,j}$, where, following the notation from Section 2.2, $n^\star_{k,j} := \frac{1}{|\Lambda_j|}\int_{\Lambda_j} n^\star(t_k, x)\,\mathrm{d}x$, and the noise is modeled as multiplicative lognormal with $\log(\varepsilon_{k,j}) \sim \mathcal{N}(0, \sigma^2)$. Our goal is to approximate the true total population

$$N^\star(t_k) := \int_\Omega n^\star(t_k, x)\,\mathrm{d}x$$

using the noisy measurements $n_{k,j}$.

We begin by observing that the expectation of the bias in the calculation is given by

$$\mathbb{E}\left[\sum_{j=1}^{J} n_{k,j} - \sum_{j=1}^{J} n^\star_{k,j}\right] = \left(\exp\left(\frac{\sigma^2}{2}\right) - 1\right)\sum_{j=1}^{J} n^\star_{k,j}.$$

This observation allows us to define an unbiased estimator for the total population when σ is known:

$$N_k := \frac{1}{\exp(\sigma^2/2)}\sum_{j=1}^{J} n_{k,j}.$$

In practice, however, σ is typically unknown and must be estimated from the data. For this purpose, we employ a loess (locally estimated scatterplot smoothing) polynomial fit applied to the logarithm of the noisy density values. Specifically, we estimate σ^2 by computing the variance of the log-transformed data around the loess estimate:

$$\tilde{\sigma}^2 := \frac{1}{KJ}\sum_{k=1}^{K}\sum_{j=1}^{J}\left(\log(n_{k,j}) - \widehat{\log(n_{k,j})}\right)^2,$$

where $\widehat{\log(n_{k,j})}$ denotes the loess-fitted value for $\log(n_{k,j})$. For precise details on the Loess fitting strategy, we point the reader to [49].
